# Pain and swelling after periapical surgery related to the hemostatic agent used: Anesthetic solution with vasoconstrictor or aluminum chloride

**DOI:** 10.4317/medoral.17782

**Published:** 2012-02-09

**Authors:** Maria Peñarrocha-Diago, Laura Maestre-Ferrín, David Peñarrocha-Oltra, Cosme Gay-Escoda, Tomas von-Arx, Miguel Peñarrocha-Diago

**Affiliations:** 1 Associate Professor of Oral Surgery. Master of Oral Surgery and Implantology. Valencia University Medical and Dental School, Valencia, Spain; 2 Master of Oral Surgery and Implantology. Valencia University Medical and Dental School, Valencia, Spain; 3 Student of Master of Oral Surgery and Implantology. Valencia University Medical and Dental School, Valencia, Spain; 4 Chairman and Professor of Oral and Maxillofacial Surgery, Director of the Master of Oral Surgery and Implantology, Barcelona University Medical and Dental School, Barcelona, Spain. Coordinator of IDIBELL Institute; 5 Vice Chairman and Associate Professor. Department of Oral Surgery and Stomatology. School of Dental Medicine, University of Bern. Switzerland; 6 Professor of Oral Surgery. Director of the Master of Oral Surgery and Implantology. Valencia University Medical and Dental School, Valencia, Spain. Investigator of IDIBELL Institute

## Abstract

Objective: To assess pain and swelling in the first 7 days after periapical surgery and their relationship with the agent used for bleeding control. Study Design: A prospective study was conducted between October 2006 and March 2009. Patients subjected to root surgery, who completed the questionnaire and who consented to the postoperative instructions were included in the study. The subjects were divided into two groups according to the hemostatic agent used: A) gauze impregnated with anesthetic solution with vasoconstrictor; or B) aluminum chloride. The patients were administered a questionnaire, and were asked to record the severity of their pain and swelling on a plain horizontal visual analog scale (VAS). Data were recorded by the patients on the first 7 postoperative days. In addition, the patients were asked to record analgesic consumption. Results: A total of 76 questionnaires (34 in group A and 42 in group B) were taken to be correctly completed. Pain was reported to be most intense two hours after surgery. At this point 52.6% of the patients had no pain. Seventy-five percent of the patients consumed analgesics in the first 24 hours. There were no significant differences between the two groups in terms of the intensity of pain or in the consumption of analgesics. Swelling reached its maximum peak on the second day; at this point, 60.6% of the patients suffered mild or moderate swelling. The Expasyl™ group showed significantly greater swelling than the gauzes group. Conclusion: The type of hemostatic agent used did not influence either the degree of pain or the need for analgesia among the patients in this study. However, the patients belonging to the Expasyl™ group suffered greater swelling than the patients treated with gauzes impregnated with anesthetic solution with vasoconstrictor.

** Key words:**Hemostasis, periradicular surgery, aluminum chloride, pain, swelling.

## Introduction

In periapical surgery, as in any other surgical procedure, side effects such as pain and swelling may occur (1-2). A number of articles have been published on the postoperative findings in periapical surgery (1-10); however, the relationship between pain and swelling and the hemostatic agent used during the surgical procedure has not been studied to date.

Kvist and Reit (4), in a study on decision-making in endodontics, compared pain and swelling after surgical versus nonsurgical retreatment. High pain scores were most frequent on the day after surgery, and swelling likewise reached its maximum level on the first postsurgical day, followed by a progressive decrease in frequency and magnitude. Chong and Pitt Ford (9) evaluated pain experience following root-end resection and filling with MTA or IRM, and concluded that there was no significant difference in the pain experienced by both treatment groups. The postoperative pain was of a relatively short duration, and its maximum intensity was recorded early in the postoperative period but progressively decreased over time. Peñarrocha et al. (1), in 60 patients subjected to periapical surgery with ultrasound and retrograde filling with silver amalgam, found the greatest prevalence of maximum-intensity pain to be recorded during the first two postoperative days; however, at that moment, two thirds of the patients had suffered no pain or mild pain. Likewise, swelling peaked on the second postoperative day, when two thirds of the patient simple showed moderate swelling. García et al. (2) related pain and swelling after periapical surgery to oral hygiene and smoking; they found that patients with poor oral hygiene before surgery presented greater pain and swelling during the first post surgical hours, and smokers before surgery also suffered more pain.

Various materials and techniques have been described for bleeding control during periradicular surgery. Von Arx et al. (11) introduced the use of Expasyl™, a paste containing aluminum chloride and kaolin and commonly used to produce gingival retraction (12,13). In an experimental study, these same authors (11) compared the hemostatic efficacy and tissue reactions of bone wax, ferric sulfate, Expasyl™ and a combination of Expasyl™ and ferric sulfate. Expasyl™ alone or in combination with ferric sulfate appeared to be the most efficient agent, and the inflammatory tissue reactions were limited to the bone defects, never extending into the surrounding tissues. In a similar study, Jensen et al. (14) found that the foreign body reactions produced in the presence of Expasyl™ and ferric sulfate did not occur if the bone cavity was refreshed with rotary instruments and irrigation.

The objective of the present study was to assess pain and swelling in the first 7 days after periapical surgery, and their relationship with the hemostatic agent used.

## Material and methods

Study sample

A total of 96 patients were treated with periapical surgery between October 2006 and March 2009, using ultrasonic retrograde cavity preparation and MTA as retrograde filling material. A retrospective study was performed of patients who completed a postoperative pain and swelling questionnaire and followed the instructions for postoperative care. The study was approved by the Local Ethics Committee and all patients signed an informed consent form.

As hemostatic material we used gauzes impregnated in anesthetic solution with vasoconstrictor among the patients treated between October 2006 and December 2007, while from January 2008 onwards aluminum chloride was used (Expasyl™, Produits Dentaires Pierre Rolland, Merignac, France). The patients were divided into two groups depending on the hemostatic agent used: A) gauze impregnated in anesthetic solution with vasoconstrictor (4% articaine and adrenalin 1:100,000); or B) aluminum chloride (Expasyl™).

Surgical procedure

All the operations were carried out by the same surgeon (MPD). Use was made of locoregional and infiltrating anesthetic techniques with 4% articaine and adrenalin 1:100,000 (Inibsa, Lliça of Vall, Barcelona, Spain). Full or partial Neumann flaps were raised, and ostectomy was carried out with a 0.27 mm round tungsten carbide drill (Jota, Switzerland) and abundant irrigation with saline solution. We performed the minimum apical resection needed to gain access to the apexes of the teeth, followed by apical curettage. The cavity was prepared for retrograde filling with a Piezon Master® ultrasound device (EMS, Electro Medical Systems S.A., Switzerland). To facilitate the procedure, a Medi Pack Pal endoscope was used (Farol Store and Co., Tuttlingen, Germany), together with a Moeller® Dental 300 surgical microscope (Möller-Wedel International, Bedel, Germany). To dry and control bleeding in the bone cavity, use was made in group A of small sterile gauzes impregnated in anesthetic solution with vasoconstrictor at a concentration of 1:100,000 (Inibsa, Lliça of Vall, Barcelona, Spain), while ExpasylTM was used in group B (Expasyl™, Produits Dentaires Pierre Rolland, Merignac, France), applied during two minutes. Lastly, the Mineral Trioxide Aggregate (MTA) filling material (ProRoot® Dentsply, USA) was prepared, inserted and condensed, following the instructions of the manufacturer. In the cases where Expasyl™ was applied, the bone crypt was refreshed with a round drill and abundant irrigation before suturing. The latter in turn was carried out using non-re absorbable Tevdek® suture material (Deknatel®, Teleflex®, Athlone, Ireland) made of polytetrafluoroethylene (PTFE) coated polyester fibers.

The same medication was prescribed in all cases during the postoperative period: amoxicillin 500 mg with clavulanic acid 125 mg every 8 hours for 7 days; ibuprofen 600 mg every 8 hours for 4 days; 0.12% chlorhexidine digluconate 3 times a day for 7 days; and paracetamol 500 mg upon demand in the event of intense pain.

Data collection

A clinical history was compiled on all patients, following a previously established protocol, recording the personal data of interest, and obtaining a detailed and orderly registry of all the pertinent clinical and radiological data, as well as of the pre-, intra- and postoperative characteristics of the patients.

Patients were given a questionnaire and an explanation on how to complete it. According to Chong and Pitt Ford (9), and in relation to pain, the patients were asked to record the severity of their pain on a plain horizontal visual analog scale (VAS), standardized to 100 mm. The phrases “no discomfort” and “intense pain” formed the left and right boundaries of the scale, respectively. Swelling in turn was recorded by the patient with another VAS. In this case the 100 mm horizontal line was divided into 10 equidistant segments serving as reference for scoring the degree or grade of swelling. In order to ensure maximum homogeneity in the patient registered scores, the following scoring system was used: 0 = absence of swelling; 1-3 = mild swelling, located within the mouth in the surgical zone; 4-6 = moderate swelling, located within the mouth and with mild swelling also outside the mouth; 7-9 = intense swelling outside the mouth in the surgical zone; and 10 = very intense extra oral swelling extending beyond the surgical zone.

The data for pain and swelling were recorded by the patient 2, 4, 6 and 12 hours after surgery, and on each day during the first 7 postoperative days. In addition, the patients were asked to record analgesic consumption. The questionnaire was to be returned on occasion of the visit for removal of the sutures.

Statistical analysis

The SPSS version 15 statistical package (SPSS, Chicago, IL, USA) was used for the statistical analysis. The data were examined at two levels: 1) descriptive statistics (mean, range, frequencies and percentages); and 2) analysis of variance (ANOVA) to determine relationships among variables, with a significance level of 0.05.

## Results

Ninety six patients were treated. As hemostatic agent we used gauzes impregnated in anesthetic solution with vasoconstrictor in 46 patients and Expasyl™ in 50. A total of 76 questionnaires (34 in the gauzes with vasoconstrictor group and 42 in the Expasyl™ group) were taken to be correctly completed. Others were not returned or were excluded if the writing was illegible or the information entered was incomplete.

The study comprised 31 males (15 in the gauze group and 16 in the Expasyl™ group) and 45 females (19 in the gauze group and 26 in the Expasyl™). The mean age in the gauze group was 40 years (±10.9), versus 43.3 years (±14.4) in the Expasyl™ group. There were no significant differences in distribution by either gender (χ2 = 0.20; p = 0.65) or mean age (t = -0.9; p = 0.38) in the patients of each group. Likewise, there were no significant differences in the symptoms of the patients in each group before surgery (χ2 = 1.53; p = 0.68) ( Table 1) ( Table 2) reports the results relating to the number of teeth, roots and canals treated.

**Table 1:**
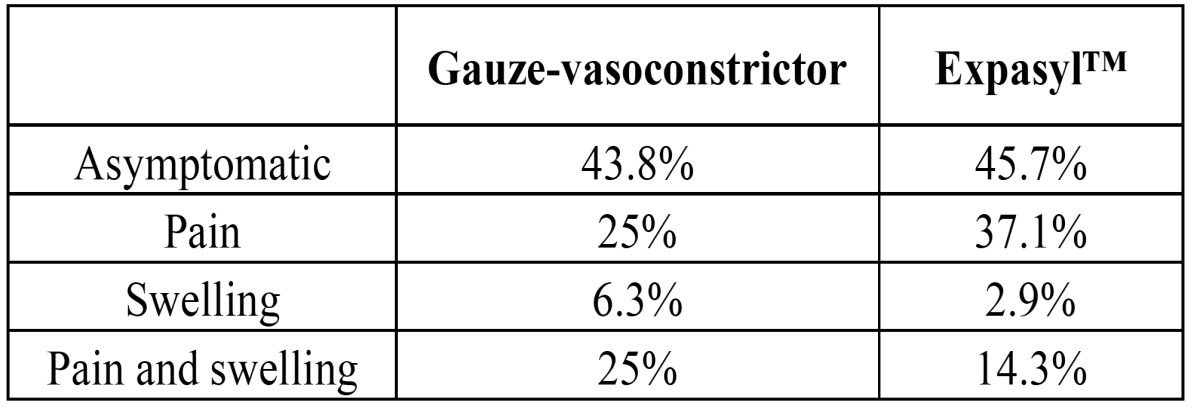
Preoperative symptoms in each patient group.

**Table 2:**
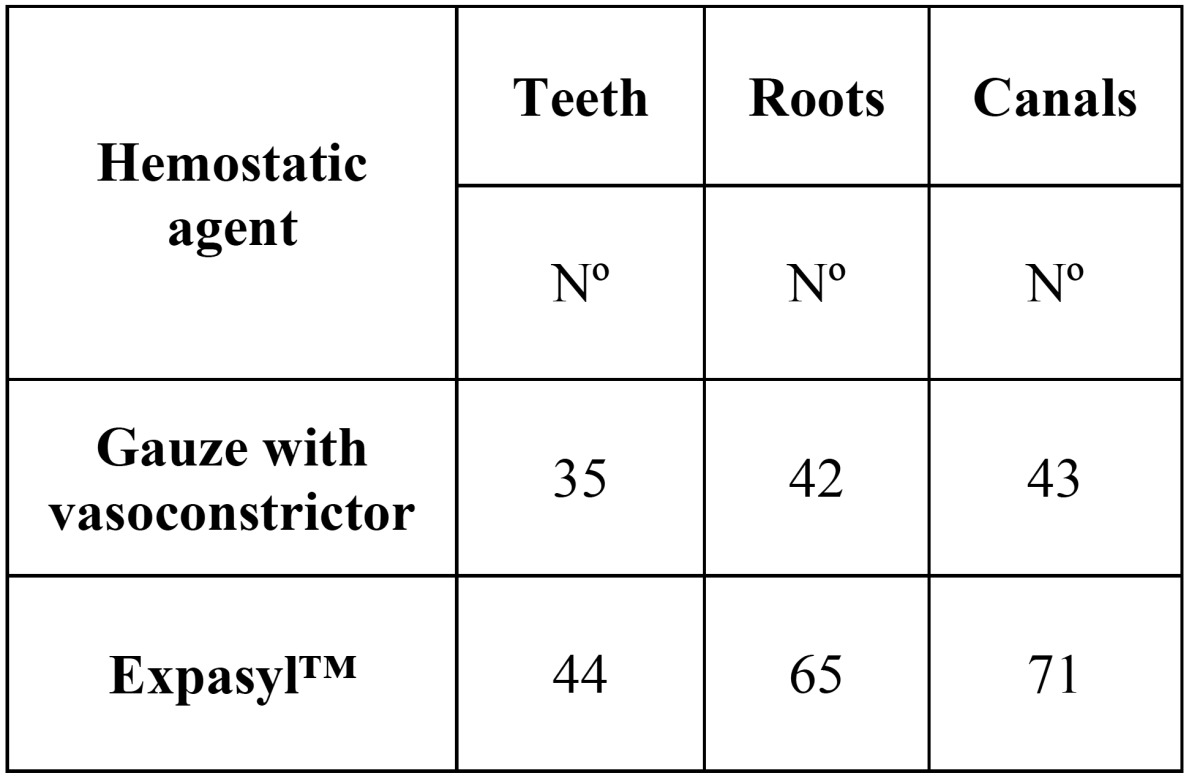
Number of teeth, roots and canals operated upon in each group, and evaluation of the diameter and area of the periapical lesions.

**Figure 1:**
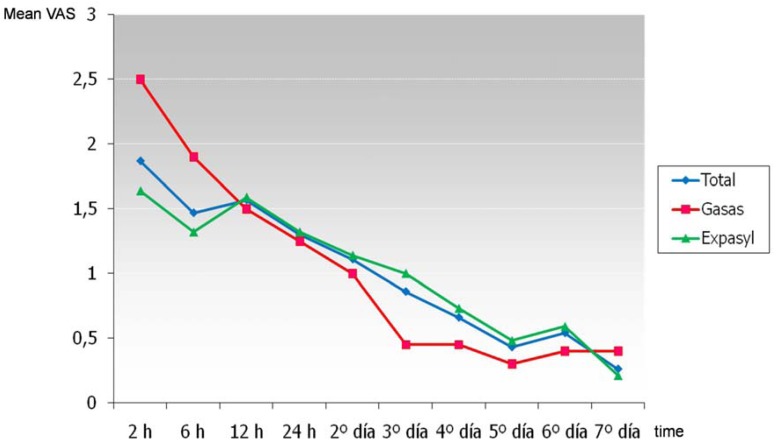
Evolution of pain over time for the global patient series and for each type of hemostatic material.

**Figure 2:**
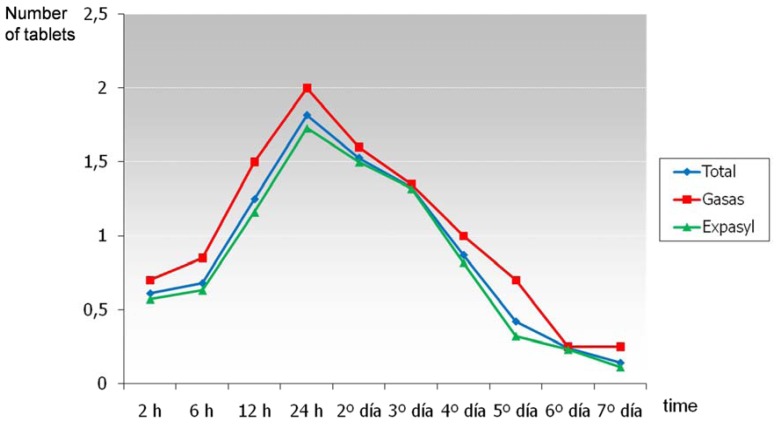
Evolution of analgesic use over time for the global patient series and for each type of hemostatic material.

Pain was reported to be most intense two hours after surgery, coinciding with the end of the anesthetic effect. At that moment, 52.6% of patients had no pain (45% in the gauze group and 55.4% in the Expasyl™ group), and posteriorly the VAS pain scores gradually decreased over time (Fig. 1). The mean pain intensity score two hours after surgery was 1.9 over 10 (2.5 in the gauze group and 1.6 in the Expasyl™ group). There were no significant differences in pain intensity between the two groups (F = 0.00; p = 0.97). The gauze group experienced greater pain at the start (at the first two registry time points), but posteriorly the pain intensity decreased faster than in the Expasyl™ group – though the differences in pain evolution over time between the two groups were not significant (F = 1.7; p = 0.16) (Fig. 1). Analgesic consumption was maximum 24 hours after surgery (Fig. 2). At this point, 75% of the total patients had used such medication, with a mean consumption of 1.8 analgesics per patient in these first 24 hours. In the gauze with vasoconstrictor group, 80% of the patients consumed analgesics in the first 24 hours, with a mean consumption of 2 analgesics per patient; in the Expasyl™ group, 73.2% of the patients consumed analgesics in the first 24 hours, with a mean consumption of 1.7 analgesics per patient. The differences in analgesic use between the two groups was not significant (F = 0.95; p = 0.33), though the patients in the gauze group showed greater consumption at all measurement time points (Fig. 2). The differences in the evolution of analgesic consumption between the two groups likewise lacked statistical significance (F = 0.45; p = 0.71). The analgesia needs were maximum in the first 24 hours, and posteriorly decreased gradually to the point where on the third postoperative day over half of the patients required no analgesia, and on day 7 the great majority (90.8%) required no such medication.

**Figure 3:**
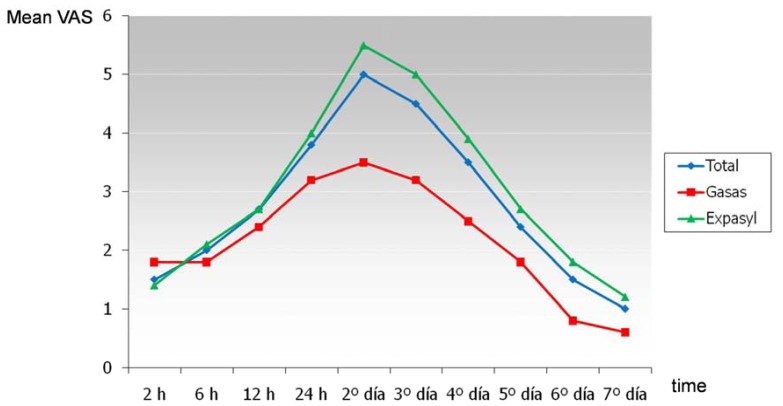
Evolution of swelling over time for the global patient series and for each type of hemostatic material.

Swelling increased progressively after surgery, reaching a maximum peak on the second day (Fig. 3).

At this point, 60.6% of the total patients presented swelling of grade 5 or less – with a mean grade of 5. In the gauze with vasoconstrictor group, 85% of the patients suffered swelling of grade 5 or lower (mean 3.6), while in the Expasyl™ group 51.9% of the patients presented swelling of grade 5 or lower (mean 5.5). The differences in the degree of swelling between the two groups were significant (F = 5.87; p = 0.02): the Expasyl™ group suffered greater swelling than the gauze group at all recording time points except the first. The differences in the evolution of swelling between the two groups was also significant (F = 3.97; p = 0.00). In this sense, swelling increased similarly in both groups up until 12 hours after surgery, though from this point onwards the increase was greater in the Expasyl™ group until the maximum peak was reached on the second day. The decrease in swelling observed after this point was faster in the Expasyl™ group up until day 5, and posteriorly the slopes for both groups proved similar – though with higher values in the Expasyl™ group (Fig. 3).

## Discussion

We obtained a total of 76 correctly completed pain and swelling questionnaires. Chong and Pitt Ford (9) underscored the difficulty of securing patient collaboration when conducting clinical studies. Twenty-four percent of their patients either failed to return the questionnaire, or the questionnaire was incomplete; in the present study 20.8% of the patients failed to return the questionnaire. In the study published by Kreisler et al. (6), 30% of the questionnaires were lost, and in the series of Iqbal et al. (15) this figure reached 44%.

In our study, pain after periapical surgery was mild and short-lasting, reaching a maximum peak two hours after the operation in both patient groups. A number of authors have likewise identified maximum pain intensity in the immediate postoperative period, coinciding with the wearing off of the anesthetic effect. In the study of Kvist and Reit (4), maximum pain was recorded on the day of surgery at night; in the studies published by Chong and Pitt Ford (9), and by Christiansen et al. (16), maximum pain was recorded 3-5 hours after surgery; and in the studies of Lin et al. (10) and Iqbal et al. (15), maximum pain intensity was recorded on the same day of the operation. In contrast, other authors have found maximum pain intensity to occur on the day after surgery (5,7,8), or 48 hours after the operation (1,2). In our series, the mean VAS score corresponding to maximum pain intensity was 1.9 over 10 (2.5 in the gauze group and 1.6 in the Expasyl™ group). Other authors have recorded slightly higher levels: Kvist and Reit (4) obtained a VAS score of 30 over 100; Lin et al. (10) recorded a score of 3.8 over 10; Iqbal et al. (15) a score of 3.62 over 10; and Christiansen et al. (16) a score of 29 over 100. In contrast, Del Fabbro et al. (17) recorded scores of more than 80 over 100 in their two patient groups. In the present study, the maximum pain intensity was recorded two hours after surgery, though at this point 52.6% of the patients experienced no pain. Tsesis et al. (5) found 76.4% of their patients to be free of pain after 24 hours. This percentage is greater than the values reported by other authors, probably because of the administration of dexamethasone before and after surgery. Lin et al. (10) found 41.8% of their patients to be without pain or with only very mild pain, while Peñarrocha et al. (1) found 33.6% of their patients to be free of pain on the first postoperative day. In comparison, almost all the patients of Kvist and Reit (4) experienced pain on the day after surgery, and Chong and Pitt Ford (9) found 90% of their patients to have suffered pain at some time during the postoperative period (comprising an interval of 48 hours).

The analgesia needs were maximum in the first 24 hours, and 75% of our patients consumed analgesics. The literature offers similar results: 67% of the patients in the study published by Kvist and Reit (4) consumed analgesics, while in one of the studies of Tsesis et al. (5) two-thirds of the patients required such medication, and in another study by these same authors the proportion reached 81% (8). The percentages reported by Chong and Pitt Ford (9) and García et al. (2) in turn were 63% and 58%, respectively.

Swelling reached a maximum 48 hours after surgery. At that point, 60.6% of the patients suffered swelling of grade 5 or lower (mild or moderate). According to some authors, swelling is maximum 24 hours after surgery (4,15,16), while others coincide with our own findings and point to the second postoperative day as the time of maximum swelling (1,2). In 2003, Tsesis et al. (5) found 64.7% of their patients to be free of swelling on the day after surgery – probably as a result of the administration of dexamethasone before and after the operation. Two-thirds of the patients evaluated by Peñarrocha et al. (1) suffered moderate swelling 48 hours after surgery. In the present study, the mean VAS score at the time of maximum swelling was 5 over 10 (3.6 in the gauze group and 5.5 in the Expasyl™ group). Similar results have been published by other authors: Kvist and Reit (4) recorded a score of 46 over 100; Iqbal et al. (15) a score of 4.7 over 10; and Christiansen et al. (16) a score of 41 over 100.

No studies have been published on the relationship between the hemostatic material used and postoperative pain and swelling following periapical surgery. However, other factors related to the surgical technique have been studied. In this sense, Tsesis et al. (8) compared the conventional rotary technique with microscope-assisted ultrasound; the patients in the latter group experienced comparatively less pain and required fewer analgesics. Chong and Pitt Ford (9) found no differences in the degree of pain or in the consumption of analgesics in the postoperative period in relation to the retrograde filling material used (IRM or MTA). According to Del Fabbro et al. (17), in those cases where incision was carried out at the base of the papilla a faster reduction in pain, swelling and analgesic consumption was noted than when using an intrasulcular incision. In the present study the differences in the degree of pain and in the consumption of analgesics between the two groups were not significant, though swelling was more pronounced in the Expasyl™ group. Aluminum chloride has been found to cause tissue inflammatory reactions when used as a gingival retraction agent. In a number of studies it produced greater swelling than the other comparator retraction techniques (18-20). However, the histological evaluation made by von Arx et al. (11) after the use of different hemostatic agents applied in the rabbit cranium showed the tissue inflammatory reactions produced after the use of Expasyl™ to be limited to the bone defects – never spreading to the surrounding tissues. Thus, the differences in postoperative swelling between the two groups in our study may be related to other factors, since the hemostatic agent was not chosen at random. Randomized, controlled clinical trials are needed to further determine the influence of hemostatic agents upon the postoperative patient symptoms (21).

## Conclusion

The type of hemostatic agent used did not influence either the degree of pain or the need for analgesia among the patients in this study. However, the patients belonging to the Expasyl™ group suffered greater swelling than the patients treated with gauzes impregnated with anesthetic solution with vasoconstrictor.
